# METABOLOMIC SIGNATURES OF HEALTHY AGING AMONG COMMUNITY-DWELLING OLDER ADULTS

**DOI:** 10.1093/geroni/igad104.2770

**Published:** 2023-12-21

**Authors:** Shanshan Yao, Megan Marron, Anne Newman, Ravi Shah, Venkatesh Murthy

**Affiliations:** University of Pittsburgh School of Public Health, Pittsburgh, Pennsylvania, United States; University of Pittsburgh School of Public Health, Pittsburgh, Pennsylvania, United States; University of Pittsburgh School of Public Health, Pittsburgh, Pennsylvania, United States; Vanderbilt University Medical Center, Nashville, Tennessee, United States; University of Michigan, Ann Arbor, Michigan, United States

## Abstract

Metabolomic signatures of healthy aging were created by relating comprehensive mass-spectrometry-based metabolomic profiles with the Healthy Aging Index (HAI), a composite of cardiovascular, lung, cognitive, metabolic, and renal function, scored 0-10. Black and white older adults (N=2017, mean age 75 years; 35% Black) in the Health, Aging, and Body Composition Study were randomly divided into a training (70%) and test (30%) sample. In the training sample, metabolites associated with baseline HAI were identified. Penalized regression was used to generate a parsimonious metabolomic risk score for baseline HAI, which was entered into linear regression models for Year 10 HAI and Cox models for mortality adjusting for baseline age, race, gender, and study site. We identified 12 baseline HAI-related metabolites associated with Year 10 HAI after adjusting for baseline HAI. One standard deviation (SD) higher metabolomic risk score was associated with 0.25 [95%confidence interval: 0.10-0.40] higher Year 10 HAI (training adjusted beta: 0.25 [0.10-0.40]; test: 0.42 [0.15-0.68]). Baseline HAI was associated with mortality (training adjusted hazard ratio: 1.54 [1.43-1.66] per SD; test: 1.33 [1.18-1.50]). Adjusting additionally for metabolomic risk score attenuated the association between baseline HAI and mortality by 41% (training) and 67% (test). In this model, the metabolomic risk score was associated with higher mortality (training: 1.28 [1.18-1.40]; test: 1.42 [1.22-1.65]). Pathway analysis suggested that metabolic pathways of Aminoacyl-tRNA biosynthesis, Arginine biosynthesis, and Citrate cycle may contribute to healthy aging. Metabolomic signatures of baseline healthy aging are associated with long-term healthy aging and mortality independent of lifestyle and traditional risk factors.

